# Comparative Transcriptomics Study of Curcumin and Conventional Therapies in Translocation, Clear Cell, and Papillary Renal Cell Carcinoma Subtypes

**DOI:** 10.3390/ijms26136161

**Published:** 2025-06-26

**Authors:** Moses Owoicho Abah, Deborah Oganya Ogenyi, Angelina V. Zhilenkova, Freddy Elad Essogmo, Ikenna Kingsley Uchendu, Yvan Sinclair Ngaha Tchawe, Akaye Madu Pascal, Natalia M. Nikitina, Onoja Solomon Oloche, Maria Pavliv, Alexander S. Rusanov, Varvara D. Sanikovich, Yuliya N. Pirogova, Leonid N. Bagmet, Aleksandra V. Moiseeva, Marina I. Sekacheva

**Affiliations:** 1 World-Class Research Center “Digital Biodesign and Personalized Healthcare”, Sechenov First Moscow State Medical University, Moscow 19991, Russia; ogenyideborah@gmail.com (D.O.O.); av.zhilenkova@gmail.com (A.V.Z.); essogmo_f@student.sechenov.ru (F.E.E.); uchenduikenna1@gmail.com (I.K.U.); ngakhatchave_i@student.sechenov.ru (Y.S.N.T.); akayepaschal48@gmail.com (A.M.P.); nikitinanm@gmail.com (N.M.N.); solomonoloche36@gmail.com (O.S.O.); alexrus146@yandex.ru (A.S.R.); v8varvara@gmail.com (V.D.S.); pirogova_yuliya96@mail.ru (Y.N.P.); bagmetln@mail.ru (L.N.B.); moiseeva_a_v@student.sechenov.ru (A.V.M.); sekacheva_m_i@staff.sechenov.ru (M.I.S.); 2 Department of Cancer Bioinformatics and Molecular Biology, Royal Society of Clinical and Academic Researchers (ROSCAR) International, Abuja 900104, Nigeria; 3 Medical Laboratory Science Department, Faculty of Health Science and Technology, College of Medicine, University of Nigeria, Enugu Campus, Enugu 410001, Nigeria; 4 Department of Public Health, James Lind Institute, Rue de la Cité 1, 1204 Geneva, Switzerland; 5 Department of General Medicine, I.M Sechenov First Moscow State Medical University, Moscow 19991, Russia; marya.pavliv@yandex.ru

**Keywords:** kidney renal clear cell carcinoma, kidney renal papillary cell carcinoma, curcumin, turmeric, tRCC, transcriptomics

## Abstract

Currently, there is no standard treatment for renal cell carcinoma (RCC) that is free of side effects and resistance. Additionally, limited information exists on how curcumin affects the gene expression profiles of patients with translocation renal cell carcinoma (tRCC) and papillary renal cell carcinoma (pRCC). The pathways responsible for metastasis in tRCC are still not well understood, and there is no established treatment or reliable biomarker to predict outcomes for metastatic tRCC. Primary clinical data from patients were retrieved from the TCGA database and analyzed using cBioPortal, stitch, string, R and Python. Various analyses were performed, including differential gene expression, protein-protein interaction (PPI) network analysis, drug-targeted gene analysis, gene ontology (GO), enrichment analyses, and systematic searches to assess the impact of curcumin on the transcriptomic profiles of tRCC, pRCC, and clear cell renal cell carcinoma (ccRCC). No significant impact of sensitive genes on survival in KIRC and KIRP was found, though a trend suggested they may delay disease progression. The combination of curcumin with sunitinib showed promise in overcoming drug resistance in ccRCC by inducing ferroptosis, reducing iron, and increasing ADAMTS18 expression. This study, leveraging data from the TCGA database and other databases explored the impact of curcumin on transcriptomic profiles in tRCC, pRCC, and clear cell RCC (ccRCC). Gene analysis revealed immune and metabolic differences, with KIRC showing a stronger immune response. This study is the first to propose that future research into the miR-148/ADAMTS18 genes and the ferroptosis pathway in tRCC and pRCC could lead to the development of new therapies and the identification of novel therapeutic targets, potentially overcoming drug resistance and metastasis.

## 1. Introduction

Renal cell carcinoma (RCC) is a type of cancer that develops in the tubules of the kidneys, which are responsible for filtering waste and reabsorbing vital nutrients. The kidneys also regulate blood pressure. RCC is the most common kidney cancer in adults, usually affecting people over 50 years of age. Risk factors include smoking, obesity, high blood pressure, and genetic conditions. Early RCC often has no symptoms, making it difficult to detect. When symptoms appear, they may include blood in the urine, side or back pain, weight loss, fatigue, and a lump in the abdomen. Early detection is crucial since RCC can spread to other organs. Advances in imaging, targeted therapies, and surgery have improved patient outcomes.

Renal tumors are classified by their cell types and locations. Papillary urothelial carcinomas develop in the ureter and pelvis, while renal medullary and collecting duct carcinomas form in the kidney medulla [1]. RCC has subtypes, including chromophobe renal cell carcinoma (chRCC) or kidney chromophobe (KICH), papillary renal cell carcinoma (pRCC) or kidney renal papillary cell carcinoma (KIRP), and clear cell renal cell carcinoma (ccRCC) or kidney renal clear cell carcinoma ((KIRC) [2]. chRCC is rare (5% of RCC cases), pRCC accounts for 10–15%, and ccRCC is the most common (70%) [3,4]. Understanding these subtypes helps with diagnosis and treatment. The World Health Organization classified RCC, but some rare subtypes remain unclassified [3,4,5]. These include renal medullary carcinoma, translocation carcinoma, tubulopapillary RCC, multilocular cystic RCC, and benign angiomyolipoma. Among non-clear cell RCCs (nccRCC), pRCC, chRCC, and translocation RCC (tRCC) are the most common [5]. Expanding knowledge of these subtypes improves patient care and research efforts.

tRCC is a rare RCC subtype with limited genetic data. It accounts for 15% of RCC cases in patients under 40, with an incidence of 1–6%. It is identified by morphology and TFE3 expression and has diverse translocations involving TFE3 and TFEB genes, leading to gene fusion within the MiTF family [6]. ccRCC is linked to VHL gene mutations, which promote tumor growth by activating VEGF and HIF [3,4,5,6,7]. pRCC has two types: type 1, which is slow-growing, and type 2, which is aggressive [8,9,10]. Both types have MET mutations, but type 2 also involves SETD2, CDKN2A, EGFR, NF2, and TERT mutations, affecting cell cycle regulation. chRCC rarely spreads but has mitochondrial changes, p53 mutations, and mTOR pathway activation [11,12,13,14]. Collecting duct carcinoma is highly immunogenic and marked by lymphocyte infiltration [15,16,17,18,19]. Renal medullary carcinoma often lacks the SMARCB1 gene, affecting chromatin remodeling. Hereditary leiomyomatosis renal cell carcinoma (HLRCC) is caused by Fumarate Hydratase (FH) mutations, leading to aggressive tumors [20,21,22,23]. Sarcomatoid RCC (sRCC) is linked to mutations in NF2, TP53, CDKN2A, BAP1, and ARIDIA [24,25,26,27].

Curcumin, a compound in turmeric, has shown potential in RCC treatment. It enhances drug effectiveness, reverses resistance to sunitinib, and promotes cancer cell death (apoptosis). It also makes cancer cells more sensitive to TRAIL, a protein that kills cancer cells without harming normal ones [28,29]. This study examines RCC subtypes (KIRC, KIRP, KICH, and tRCC) using TCGA clinical patients’ data. It analyzes curcumin-sensitive genes and gene expression changes after treatments with sorafenib, temsirolimus, and radiation therapy. By integrating transcriptomic data with clinical factors, the research aims to identify and compare key genes in RCC progression and treatment response. Findings of this study will enhance a better understanding of the various RCC subtypes, highlight potential drug targets, and assess the therapeutic roles of curcumin.

### Aim and Objectives

This article aims to provide comprehensive and accurate comparative transcriptomic analyses of curcumin and conventional therapies in translocation, clear cell, and papillary renal cell carcinoma subtypes of renal cell carcinomas (RCC). The objectives of this study include the following:

1. To study the associated key biomarkers and compare the gene activity patterns of translocation renal cell carcinoma (tRCC), clear cell renal cell carcinoma (ccRCC), and papillary renal cell carcinoma (pRCC), respectively, using primary clinical data of patients from the TCGA database.
2. To compare how patients respond to curcumin (turmeric) and standard treatments by looking at ways to improve patient care and develop safer and more effective personalized treatments with zero side effects.
3. To provide a comprehensive and accurate analysis of the available literature on pRCC, tRCC, ccRCC, and curcumin, shedding light on the intricate mechanisms and potential therapeutic targets in renal cancer.
4. To compare the anti-drug resistance effect of curcumin across the various RCC subtypes under study.

## 2. Results

### 2.1. Correlation Between Curcumin-Sensitive Genes and Patient Survival in KIRC and KIRP

**Figure 1:**
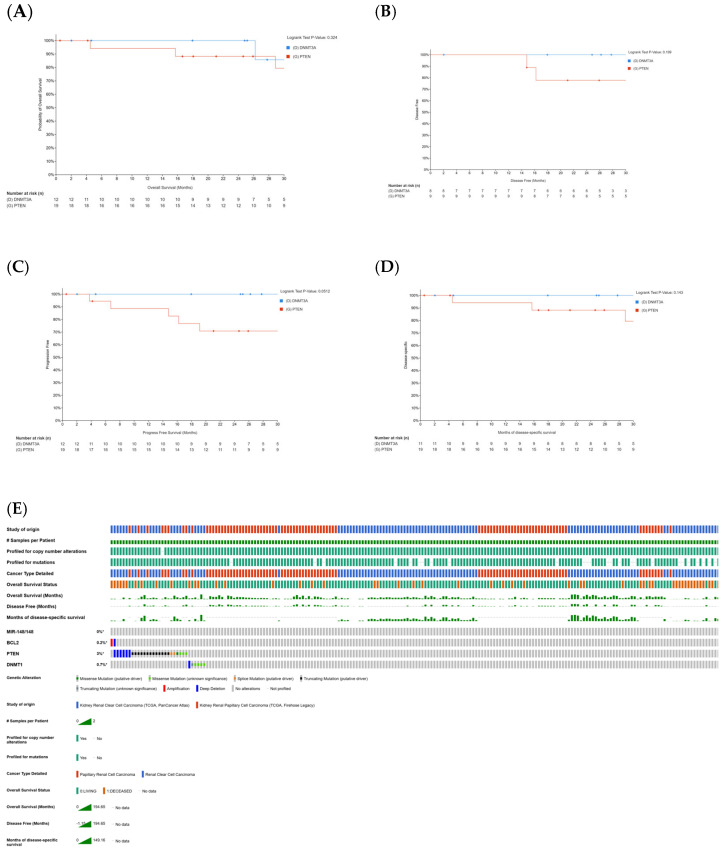
KIRC and KIRP survival data correlation with curcumin-sensitive genes. (**A**) Depicts the overall survival (OS) curve, with a log-rank *p*-value of 0.324, indicating a lack of significant correlation between patient OS and the expression of DNMT3A and PTEN. DNMT3A, a gene inhibited by curcumin, is known for its oncogenic activity, while PTEN, upregulated by curcumin, plays a crucial role in tumor suppression. (**B**) The disease-free survival (DFS) curve, with a log-rank *p*-value of 0.199, suggests no significant association between DFS and the expression of DNMT3A and PTEN. (**C**) The progression-free survival (PFS) curve, with a log-rank *p*-value of 0.0512, indicates a notable trend toward significance. This suggests that variations in DNMT3A and PTEN expression levels may have a meaningful impact on PFS. (**D**) The disease-specific survival (DSS) curve, with a log-rank *p*-value of 0.143, demonstrates the absence of a significant correlation between DSS and DNMT3A or PTEN expression. (**E**) The OncoPrint analysis reveals a higher overall survival rate in KIRP patients compared to KIRC patients, who exhibit specific genetic alterations in curcumin-sensitive genes. These alterations include 0% in MIR-148/148, 0.2% amplification and deep deletion in BCL-2, 3% deep deletion, truncating, splice, and missense mutations in PTEN, and 0.7% deep deletion and missense mutations in DNMT1. The asterisks (*) in Figure 1 highlight the differential extent of curcumin-sensitive genes altered in the study.

We analyzed the relationship between curcumin-sensitive genes (PTEN and DNMT3A) and patient survival using the Kaplan–Meier (KM) curve and log-rank test. The results showed no significant correlation between gene expression and overall survival (OS), with a *p*-value of 0.324 (Figure 1A). This means that changes in PTEN and DNMT3A expression levels have little to no effect on patient survival after being diagnosed or treated for KIRP or KIRC.

Similarly, for disease-free survival (DFS), the analysis yielded a *p*-value of 0.199 (Figure 1B), suggesting no significant link between PTEN and DNMT3A expression and the time before disease recurrence or progression. However, for progression-free survival (PFS), the *p*-value was 0.0512 (Figure 1C), which is close to statistical significance. This suggests that variations in PTEN and DNMT3A expression may influence how long a patient lives with cancer without the disease worsening after treatment.

Further analysis of disease-specific survival (DSS) resulted in a *p*-value of 0.143 (Figure 1D), indicating no significant impact of PTEN and DNMT3A expression on patient survival after treatment. Additionally, our OncoPrint tool analysis showed different survival patterns in KIRC and KIRP patients. A heatmap revealed that KIRP patients, especially those with specific genetic changes in curcumin-sensitive genes like MIR-148/148, BCL2, PTEN, and DNMT1, had a higher overall survival rate compared to KIRC patients (Figure 1E).

### 2.2. Effects of Conventional Therapies on Sensitive Genes in Renal Clear Cell Carcinoma (KIRC)

**Figure 2:**
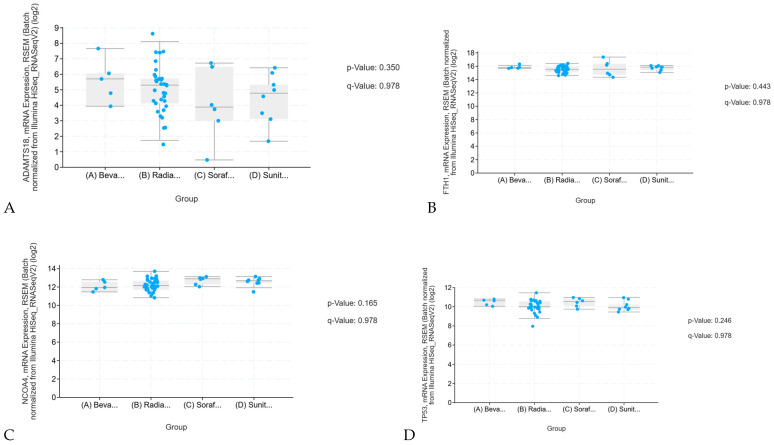
RNAseq Differential Expression of Curcumin-Sensitive Genes: This analysis examines the mRNA expression levels of curcumin-sensitive genes in relation to conventional therapies including bevacizumab, radiation, sorafenib, and sunitinib. Specifically, (**A**) presents a *p*-value of 0.350 and a q-value of 0.978 for ADAMTS18, (**B**) shows a *p*-value of 0.443 and a q-value of 0.978 for FTH1, (**C**) reports a *p*-value of 0.165 and a q-value of 0.978 for NCOA4, and (**D**) displays a *p*-value of 0.246 and a q-value of 0.978 for TP53. All *p*-values are well above the conventional threshold of 0.05, indicating no statistically significant associations between the expression of these genes and the response to conventional therapies. The elevated *p*-values suggest that any differences observed are likely due to random variation. Additionally, the consistently high q-values further confirm that, after adjusting for multiple testing, none of the results remain statistically significant and each carries a high likelihood of being a false positive. In (**A**–**D**), the abbreviations are defined as follows: Beva represents Bevacizumab, Radia denotes Radiation Therapy, Soraf corresponds to Sorafenib, and Sunit indicates Sunitinib.

The mRNA sequencing analyses of curcumin-sensitive genes including ADAMTS18, FTH1, NCOA4, and TP53 (Figure 2) show that the expression levels of these curcumin-sensitive genes are not significantly associated with patients’ response to standard treatments, including radiation therapy, bevacizumab, sorafenib, and sunitinib. The high *p*-value suggests that the result is likely due to chance and not statistically meaningful. Additionally, the very high q-value confirms that, after adjusting for multiple comparisons, the result still remains unreliable. Therefore, this finding does not suggest a true biological association and does not require further investigation.

Studies also indicate that KIRC cells resistant to sunitinib became significantly more sensitive when treated with a combination of sunitinib and curcumin, leading to significantly reduced cell growth (*p* < 0.05). Curcumin also significantly lowered ionized iron levels and decreased the expression of proteins linked to ferroptosis at a statistically significant level (*p* < 0.05). Additionally, it significantly increased the expression of ADAMTS18 (*p* < 0.05). The combined treatment of sunitinib and curcumin, analyzed using the CCK-8 assay, led to a significant reduction in the growth of the 786-O-DR ccRCC cell line, and this combination therapy significantly lowered the IC50 value of the drug-resistant 786-O-DR cell line, meaning the treatment was more effective at reducing resistance while highlighting a significant reduction in the growth of both A498-DR and 786-O-DR ccRCC cell lines, with a statistically significant difference (*p* < 0.05) observed across different treatment groups. 

#### Sunitinib Resistance in KIRC and KIRP: Validation and Therapeutic Benefit of Combination Therapy

**Figure 3:**
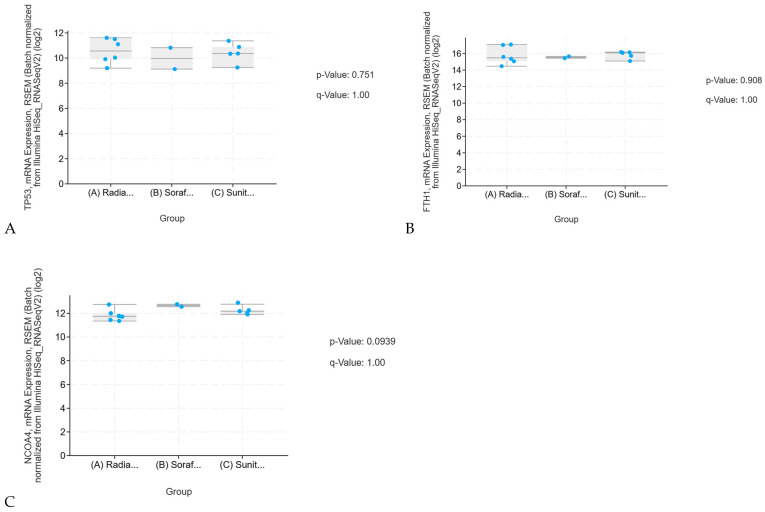
A validation study of the impact of conventional therapies on the expression of ferroptosis-related genes in KIRP and KIRC: The combined *p*- and q-values suggest that there is no statistically significant effect of KIRP and KIRC treatment therapies on the mRNA expression of TP53, NCOA4, and FTH1. The high q-values, in particular, suggest that any observed changes in gene expression could be due to random variation rather than any meaningful biological effect. Thus, we conclude that these therapies do not significantly alter the mRNA expression of these genes in both KIRP and KIRC. In (**A**–**C**), the abbreviations are defined as follows: Radia denotes Radiation Therapy, Soraf corresponds to Sorafenib, and Sunit indicates Sunitinib.

Additional RNA sequencing validation of individual genes (Figure 3) showed that radiation therapy, bevacizumab, sunitinib, and sorafenib have no significant effect on the expression of ferroptosis-related genes. This contrasts with results from Western blotting and real-time PCR, which showed that combining curcumin with sunitinib led to a significant effect with much stronger response compared to the independent use of sunitinib. Curcumin helps reduce resistance to sunitinib in KIRC by activating the ferroptosis pathway, which is important for preventing drug resistance. The combination of curcumin and sunitinib significantly lowered iron ion levels in KIRC and reduced the expression of key ferroptosis-related genes including p53, NCOA4, and FTH1. These results suggest that curcumin may inhibit both the mRNA and protein expressions of these genes, supporting its potential role in triggering ferroptosis and combating drug resistance in both KIRC and KIRP.

### 2.3. Differential Gene Expression Analysis in KIRP and KKIRC

**Figure 4:**
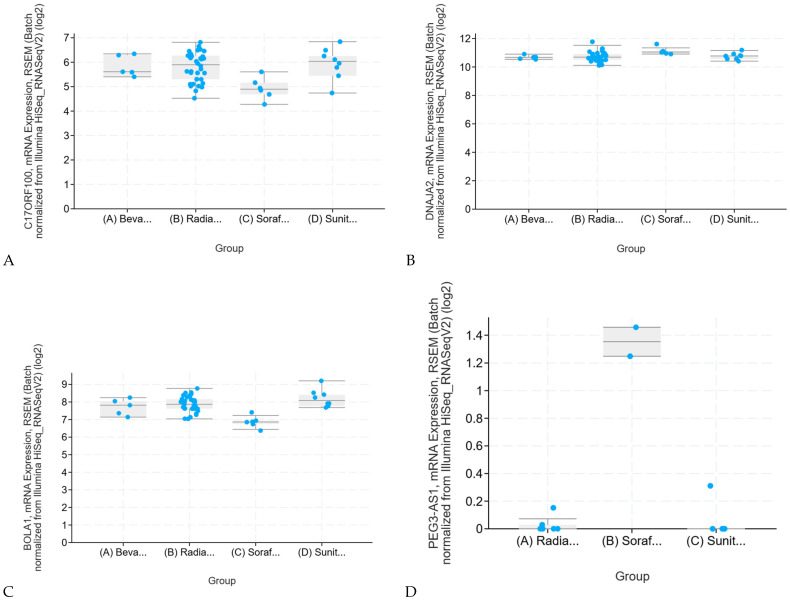
Evaluating Gene Expression Significance in RCC Subtypes: (**A**) Indicates a low *p*-value of 0.007372 which suggests a statistically significant result. However, when multiple comparisons are considered, the association between C17ORF100 mRNA expression in KIRC and response to treatments is not significant (high q-value of 0.978). After correcting for multiple tests, the result loses its statistical significance, reducing its reliability. Therefore, it is unlikely to be a true biological association and should not be pursued unless more evidence supports it. (**B**) The *p*-value of 0.0455 suggests a weak association between DNAJA2 expression and response to treatments. However, the high q-value of 0.978 shows that the result is likely a false positive and should not be pursued without additional evidence. (**C**) The low *p*-value of 0.000006855 shows a strong link between BOLA1 mRNA expression and treatment response in KIRC. While the q-value of 0.0678 suggests some risk of a false positive, the result is still meaningful and warrants further investigation. (**D**) The low *p*-value of 0.0000000897 indicates a strong association between PEG3-AS1 expression and treatment response in KIRP. Despite a slightly higher q-value of 0.0005757, the result is promising and should be further validated. In (**A**–**D**), the abbreviations are defined as follows: Beva represents Bevacizumab, Radia denotes Radiation Therapy, Soraf corresponds to Sorafenib, and Sunit indicates Sunitinib.

Differential expression analysis and gene intersection study reveals that CYP4A22, SLC25A44, PHLPP2, C17ORF100, DNAJA2, and PCAT18 showed higher expression levels in both KIRC and KIRP patients who underwent immunotherapy and radiation therapy, in contrast DAZL, UBAP2L, NTAN1, C17ORF99, EEF2KMT, and FAM74A1 had the lowest expression levels in the intersection study. Analysis of the top regulated genes shows that C17ORF100 and DNAJA2 were the only statistically significant genes (Figure 4A,B) among the upregulated genes in the intersection study of KIRP and KIRC genes, making them strong candidates for further investigation.

A more detailed analysis of each individual gene revealed that BOLA1 had the highest significant response to radiation therapy, bevacizumab, sorafenib, and sunitinib treatments in KIRC (Figure 4C), and in contrast, PEG3-AS1 showed the highest significant response to radiation therapy, sorafenib, and sunitinib, respectively (Figure 4D).

Recognizing the molecular and transcriptomic differences between KIRC and KIRP is crucial for advancing personalized treatment strategies. Insights from these variations can guide the development of targeted therapies, leading to more effective disease management and improved patient survival. Additionally, analyzing gene expression patterns in these RCC subtypes helps identify potential biomarkers for treatment, diagnosis, and prognosis. 

### 2.4. PPI Network Analysis of Key Genes in KIRC and KIRP

**Figure 5:**
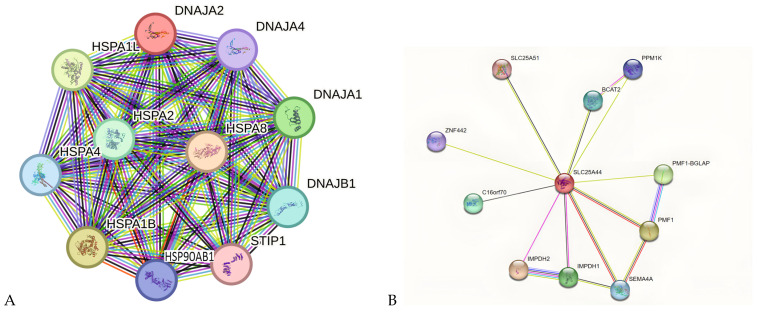

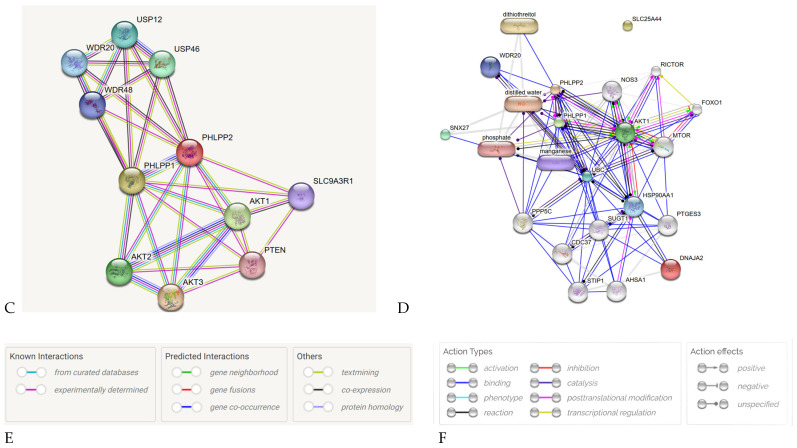
Differentially expressed gene (DEG) protein–protein interaction (PPI) analysis. The STRING Bioinformatics software identified 34 hub genes related to KIRC and KIRP, visually represented through figures showing gene relationships with lines and circles. K-means clustering grouped DNAJA2-associated genes into three distinct clusters (**A**), while SLC25A44 analysis revealed seven clusters of genes named after their contents (**B**). The PHLPP2 analysis unveiled 11 KIRP and KIRC genes divided into three clusters based on protein–protein interactions (**C**). Figure (**D**) depicts the intersection study of DNJA2, SLC25A44 and PHLPP2. (**E**) Represents the legend for Figures (**A**–**C**). (**F**) Represents the legend for Figure (**D**).

Using STRING bioinformatics software, we conducted a protein–protein interaction (PPI) network analysis, identifying 34 key genes associated with KIRC and KIRP (Figure 5). The visual representation of this network shows gene relationships, where circles represent genes and lines indicate interactions between them. To further classify these genes, K-means clustering was applied. When analyzing DNAJA2-associated genes, three distinct gene clusters were identified (Figure 5A). Similarly, the PPI network analysis of SLC25A44, combined with K-means clustering, revealed seven gene clusters linked to KIRC and KIRP (Figure 5B). Each cluster was named according to its grouped genes. Additionally, PPI analysis of PHLPP2 identified 11 related genes, which were further divided into three clusters (Figure 5C). Figure 5D illustrates the overlap of genes linked to both DNJA2 and PHLPP2, while also highlighting the absence of shared genes between DNJA2 or PHLPP2 and SLC25A44. This observation suggests the need for further investigation. These findings help categorize genes based on molecular similarities and their potential roles in KIRC and KIRP.

### 2.5. Canonical Pathway and Gene Ontology (GO) Analysis of KIRP, KIRC, and KICH 

The gene intersection analysis identified 1208 differentially expressed genes (DEGs) among KIRP, KIRC, and KICH, with 607 upregulated and 601 downregulated genes. Gene Ontology (GO) analysis revealed a complex relationship among these kidney cancer subtypes, showing that only 786 genes (27.7%) were shared across all three. The GO enrichment study highlighted the significant role of tumor-associated genes in leukocyte migration, DNA transcription, cell maturation, DNA repair, growth factor activity, cell differentiation, and cell cycle regulation. In KIRC, GO terms indicated an enhanced immune response, including cytokine secretion, T-cell activation, leukocyte differentiation, and targeted cell destruction. In contrast, KIRP exhibited a dual function in metabolic and immune response processes, including carbohydrate and lipopolysaccharide binding, mononuclear cell migration, and interleukin-1 proliferation.

Pathway analysis identified 32 common pathways among KIRC, KIRP, and KICH (kidney chromophobe carcinoma), with several uniquely dysregulated pathways, including calcium signaling, cytokine-cytokine receptor interaction, steroid hormone biosynthesis, cell adhesion molecules (CAMs), and PPAR signaling. In KIRC, dysregulated pathways were primarily associated with natural killer cell cytotoxic activity, as well as Th1, Th2, and Th17 cell proliferation, which are linked to tissue graft rejection and immune response disorders. Notably, the JAK-STAT, chemokine, and Rap-1 signaling pathways were specifically activated in KIRC, emphasizing their role in cancer progression. Additionally, KEGG pathway analysis in KIRP highlighted key pathways such as cGMP-PKG signaling, tyrosine metabolism, primary immunodeficiency, Wnt/β-catenin signaling, p53 signaling, and PI3K-Akt signaling. Further investigation into the tumor microenvironment and immune response identified 5699 DEGs in KIRC, with 3804 upregulated and 1895 downregulated genes compared to normal tissues. Validation studies identified 4896 DEGs in KIRP (2899 upregulated, 1997 downregulated) and 5759 DEGs in KICH (3074 downregulated, 2685 upregulated) compared to normal tissues [30,31,32,33,34,35,36,37,38,39,40,41,42,43,44,45,46,47,48,49,50,51,52,53,54,55,56,57,58,59,60,61,62,63,64,65,66,67,68,69,70,71,72,73,74,75,76,77,78].

### 2.6. Mutation Analysis of KIRC, KIRP and KICH

Our analysis of clinical data from the TCGA database has shown that certain genes are commonly mutated in different types of kidney cancer, which may contribute to how these cancers develop and progress. For example, in KICH (kidney chromophobe carcinoma), the genes PTEN, TP53, ZAN, MUC4, and TTN were found to have frequent mutations. These mutations are important because they play a role in how the cancer grows and spreads. Understanding these genes can help researchers and physicians develop better treatments to target these specific genetic changes. In KIRC, other genes like VHL, TTN, BAP1, PBRM1, and SETD2 showed high mutation rates. This suggests that these mutations could be important for oncogenic activities, and more research is needed to develop therapies that can specifically target these genetic alterations. By focusing on these genes, physicians may be able to improve how they treat patients with KIRC and offer more effective therapies.

**Figure 6:**
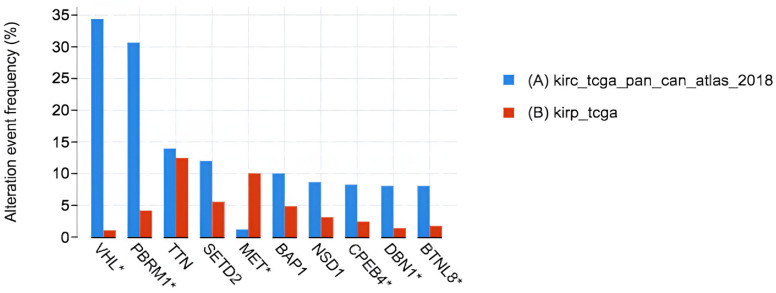
Log-rank test for the genes with the highest mutation frequencies in KIRC and KIRP.

Similarly, in KIRP, the most commonly mutated genes were MUC16, SETD2, MET, TTN, and KMT2C. These mutations also suggest that different treatments might be needed for KIRP patients. Tailoring therapies to target these specific genetic mutations could lead to better outcomes for patients, as the treatments would be more precise and effective in addressing underlying causes of the cancer. These findings are important because they emphasize the need for personalized treatment plans that take into account the specific genetic mutations in each type of kidney cancer. By targeting these mutations, physicians could improve survival rates and quality of life for kidney cancer patients. Figure 6 in the study illustrates these results, showing the different mutated genes across the kidney cancer subtypes.

This TCGA dataset provides valuable insights into the genes with the highest mutation frequencies that exhibit statistically significant differences between KIRC and KIRP (*p* < 0.05). These findings highlight crucial genetic alterations that could play a fundamental role in the initiation, development, and progression of these cancers. By identifying these key mutations, these data contribute to a deeper understanding of the molecular mechanisms underlying KIRC and KIRP, potentially aiding in the development of targeted therapeutic strategies and improved diagnostic approaches. It is evident from the data that genes such as VHL, TTN, BAP1, PBRM1, and SETD2 are consistently identified as the most frequently mutated genes in KIRC, underscoring their potential importance as key drivers of tumorigenesis in this specific cancer subtype. Similarly, this analysis reveals that MUC16, SETD2, MET, TTN, and KMT2C are among the genes exhibiting the highest frequency of mutations in KIRP, emphasizing the diverse genetic landscape and highlighting potential targets for further research and therapeutic interventions. The asterisks (*) used on VHL, PBRM1, MET, CPEB4, DBN1 and BTNL8 in Figure 6 denote the genes implicated in both KIRC and KIRP pathogenesis as shown by the analysis. 

### 2.7. Immunological Landscape of KIRC and KIRP

The immune landscape analysis using ssGSEA (single-sample Gene Set Enrichment Analysis) shows that KIRC has a stronger immune response than KIRP. T-cell marker genes are more highly expressed in KIRC, including various T-cell subtypes such as T follicular helper cells, CD4 T-cells, regulatory T-cells (Treg), CD8 T-cells, natural killer T-cells, type 1 helper T-cells, and gamma delta T-cells. Additionally, immune cells such as mast cells, immature and activated immune cells, myeloid-derived suppressor cells (MDSCs), macrophages, and neutrophils are more prevalent in KIRC compared to KIRP. KIRC also shows the highest level of T-cell exhaustion, indicated by increased activity of immune checkpoint molecules like FASLG, CTLA4, PDL1, PD1, LAG3, and TIM3, which are involved in immune suppression in cancer [79,80,81,82,83].

#### 2.7.1. Antigen Presenting Cells (APCs) in KIRP and KIRC

Dendritic cells (DCs) are more activated in KIRC than in KIRP. DC-dependent immune cells, such as IRF4-DC and BATF3-DC, are expressed more abundantly in KIRC, indicating a stronger [79,80,81,82,83] connection between APC activity, innate immunity, and KIRC. APCs, especially dendritic cells, play a key role in initiating both innate and adaptive immune responses by expressing important molecules like MHC Class 1, co-inhibitory and co-stimulatory molecules, and the Human Leukocyte Antigen (HLA) family. These molecules are more highly expressed in KIRC than in KIRP. The differentiation of precursor DCs into IRF4-DC and BATF3-DC is controlled by transcription factors IRF8 and BATF3, with IRF4 playing a crucial role in the maturation of IRF4-DC immune cells [84,85,86,87].

#### 2.7.2. Chemokine Genetic Signature Differences Between KIRP and KIRC

KIRC shows a chemokine infiltration rate that is three times higher than KIRP. KICH (chromophobe renal carcinoma) has the highest expression of CCL21 and CCL25 chemokines among the three subtypes. CXCL16 and CXCL13 play a bigger role in the immune response in KIRC than in KIRP, while CCL14 and CXCL25 are more important in KIRP. Notably, T-cells upregulate the expression of chemokines like CCL2, CCL10, CCL3, CCL5, CCL9, and CCL4, which activate their receptors on CD8+ T-cells. CCL5, CCL2, CCL4, and CCL3 are more highly expressed in KIRC compared to both KIRP and KICH. In contrast, CXCL11, CXCL9, and CXCL10 are more expressed in KICH compared to KIRP. Interestingly, CCL25 shows a weaker relationship with cytolytic T-cells, suggesting that KICH might trigger a less strong immune response than KIRC and KIRP. Furthermore, CCL20 is more highly expressed in KICH than in both KIRC and KIRP, supporting earlier research that mutations in the EGFR/Ras pathway promote tumor growth by increasing EGFR/Ras expression, which boosts the immune-activating chemokine CCL20 [88,89,90,91,92,93,94,95,96,97]. Previous studies have shown that the CXCL13/CXCR5 axis contributes to tumor growth, proliferation, metastasis, and invasion. Additionally, the CCL25-CCR9 signaling pathway is important for T-cell development and helps hematopoietic precursors migrate from the bone marrow to the thymus, which is crucial for T-cell proliferation.

#### 2.7.3. Immune Differences and Lymphocyte Activation in KIRC, KIRP, and KICH

When comparing KIRC, KIRP, and KICH to their respective healthy tissues, no significant differences in CD3+ T and B cell expression were found. However, there was a clear difference in CD8+ T-cell and cytotoxic lymphocyte levels, with KIRC showing much higher levels than its healthy tissue counterpart. No similar differences were seen between the tumor and healthy tissues of KIRP and KICH. Among the three subtypes, KICH had a much lower immune score compared to both KIRC and KIRP. This finding was confirmed by a validation study that closely matched the results from the analyzed datasets [98,99,100]. This study also found that KIRC has higher expression levels of monocytes, myeloid dendritic cells, and natural killer (NK) cells than both KIRP and KICH, supporting the broader immune differences seen between these subtypes.

#### 2.7.4. CD8+ T-Cell Infiltration in KIRC and KIRP

TCGA RNA sequencing data on immune cell infiltration, especially through the measurement of CD8A expression and mRNA levels of granzyme A (GZMA) and perforin 1 (PRF1), confirmed that CD8A expression is higher in KIRC than in KIRP. However, while CD8+ cytolytic T-cell infiltration is more significant in KIRP than in KICH, immune cell cytolytic activity (CYT) is higher in KICH than in KIRP, which needs further exploration. Gene Ontology (GO) and KEGG pathway analyses show significant differences in CD8+ T-cell infiltration between KIRC and KIRP. This variation in CD8+ T-cell recruitment, which is critical for antitumor immunity, highlights the distinct immune cell infiltration profiles of these cancer types. Combining the findings from GO and KEGG analyses with ssGSEA results reveals substantial differences in CD8+ T-cell infiltration, immune landscapes, and antigen-presenting cell activity between KIRC and KIRP [101,102,103,104,105,106,107]. These results enhance our understanding of the immunological differences between these cancers and provide valuable insights for future research and treatment development. Further investigation into these immune variations is important for creating more targeted therapies tailored to the unique immune profiles of KIRC and KIRP.

## 3. Discussion

### 3.1. Scientific Interpretation of Observed Parameters: Insights into Key Genes and Network Pathways

Figure 1C highlights the expression levels of DNMT3A and PTEN, showing their near-significant correlation with progression-free survival (PFS) in KIRC and KIRP patients. DNMT3A, an important gene involved in DNA methylation, plays different roles in KIRC and KIRP. In KIRC, DNMT3A mutations cause epigenetic changes that disrupt DNA methylation, contributing to disease progression. These mutations can also alter immune-related gene methylation in the tumor microenvironment, allowing tumor cells to evade the immune system and resist apoptosis. Interestingly, increased DNMT3A expression in KIRC is linked to enhanced tumor suppression, suggesting a potential protective role. In contrast, in KIRP, higher DNMT3A expression promotes tumor cell proliferation by silencing tumor suppressor genes, accelerating KIRP progression. DNMT3A mutations in KIRP are associated with poorer survival, highlighting its prognostic importance. Targeting the DNMT3A-DNA methylation pathway could be a promising therapeutic strategy for KIRP, and DNMT3A methylation patterns and protein/mRNA levels might serve as valuable biomarkers for diagnosis and prognosis in both KIRC and KIRP.

PTEN, a well-known tumor suppressor gene, regulates the PI3K/AKT/mTOR signaling pathway, which is crucial for tumor progression, metastasis, and angiogenesis in both KIRC and KIRP. PTEN mutations are more common in KIRC but are also found in aggressive KIRP, especially the type 2 subtype, where they drive rapid tumor growth. PTEN status could therefore be a valuable prognostic and diagnostic marker for aggressive KIRP. Targeting the PI3K/AKT/mTOR pathway in patients with low PTEN expression may offer a promising treatment option. Exploring PI3K/AKT/mTOR inhibitors in these patients could lead to improved treatment strategies for both KIRC and KIRP.

The upregulation of ADAMTS18 and downregulation of p53, NCOA4, and FTH1 in KIRC (Figure 2 and Figure 3) play important roles in tumor suppression. While both p53 and ADAMTS18 act as tumor suppressors, p53 changes have a more significant impact on KIRP progression. NCOA4 and FTH1 are involved in iron metabolism and ferroptosis, with NCOA4 acting as a tumor suppressor and FTH1 promoting tumor progression. A combined therapeutic approach using MDM2 inhibitors (to restore p53 function), epigenetic therapies (to regulate ADAMTS18), and ferroptosis activators (to enhance NCOA4-mediated suppression) could provide a promising strategy for treating KIRC and KIRP patients.

Future research into the miR-148/ADAMTS18 and ferroptosis pathways in tRCC and pRCC may lead to breakthroughs in therapy. Understanding the molecular mechanisms behind these pathways could reveal new treatment targets and improve strategies for overcoming drug resistance in renal cell carcinoma.

### 3.2. Curcumin as a Therapeutic Agent: Targeting Cancer Through Apoptosis and Cell Cycle Regulation

Curcumin has been extensively studied for its anti-cancer effects, mainly due to its ability to modulate critical molecular pathways involved in tumor growth. Studies show that curcumin induces apoptosis, a process that eliminates cancer cells and halts tumor growth. It also inhibits key signaling pathways like NF-κB, PI3K/Akt, and Wnt/β-catenin, which are involved in cancer cell survival, growth, and metastasis. In renal cell carcinoma (RCC), curcumin suppresses the PI3K/AKT pathway, which plays a crucial role in cell growth and survival. It promotes apoptosis by increasing Bcl-2 activity, which prevents cell death, while decreasing Bax gene activity, which promotes apoptosis. This balance between survival and death signals is crucial for curcumin’s anti-cancer effects. Additionally, curcumin can induce cell cycle arrest in ccRCC by inhibiting cyclin B1, a protein required for cell cycle progression. These actions make curcumin a potential therapeutic agent for targeting cancer cells in ccRCC.

Effects of curcumin have been observed in various cancers, including renal, pancreatic, breast, colon, prostate, and lung cancers, highlighting its broad potential. In renal cancer, curcumin downregulates pro-inflammatory cytokines, inhibits angiogenesis (the formation of new blood vessels in tumors), and prevents epithelial-to-mesenchymal transition (EMT), a key process in metastasis. These properties suggest that curcumin could be an effective complement to conventional cancer therapies, such as chemotherapy, radiotherapy, and immunotherapy, which often have limited success when used alone.

### 3.3. The Role of Curcumin and Temsirolimus in Renal Cancer Treatment

Curcumin, a compound found in turmeric, has shown promising results when combined with temsirolimus, a first-line treatment for advanced renal clear cell carcinoma. This combination enhances apoptosis, which is vital for stopping cancer cell growth and metastasis. Temsirolimus targets the mTOR pathway, which is important for cancer cell survival, while curcumin helps activate the p53 protein, which regulates cell death. Together, they increase YAP (Yes-Associated Protein) activity, which controls cell growth and death, making the combination more effective than either treatment alone. The combined therapy works in several ways: a. Boosting Apoptosis: By activating p53 and increasing the activity of enzymes like cleaved caspase-3 and poly (ADP-ribose) polymerase, the treatment enhances cancer cell death. b. Suppressing Survival Signals: It lowers Bcl-2 levels, a protein that helps cancer cells survive. c. Enhancing Drug Sensitivity: Pre-treating cancer cells with low doses of curcumin makes them more sensitive to temsirolimus, reducing the risk of drug resistance.

Studies suggest that YAP is crucial for curcumin’s ability to activate p53, as blocking YAP reduces curcumin’s effectiveness. Overall, the combination of curcumin and temsirolimus offers a promising strategy for treating kidney cancer, especially in cases where drug resistance is a concern [62,79,80,81,82,83,84,85,86,87,88,89,90,91,92,93,94,95,96,97,98,99,100,101,102,103,104,105,106].

### 3.4. The Combined Effect of Curcumin and Radiation Therapy

Radiation therapy is a common treatment for kidney cancer, but its effectiveness can be reduced when cancer cells develop resistance. Research shows that combining curcumin with radiation therapy significantly improves treatment outcomes by making cancer cells more responsive to radiation. This effect occurs because curcumin inhibits a gene called NF-κB, which plays a key role in cancer progression and resistance to treatment. By blocking NF-κB, curcumin disrupts multiple pathways that help cancer cells survive and spread, including the following:

a. Reducing Tumor Growth: Curcumin suppresses VEGF, a protein that promotes blood vessel formation in tumors (angiogenesis), and COX-2, an enzyme linked to inflammation and cancer progression.
b. Blocking Survival Pathways: It inhibits the PI3K/Akt and mTOR pathways, which are crucial for cancer cell survival and growth.
c. Preventing Cancer Spread: Curcumin lowers the activity of enzymes like MMP-2 and MMP-9, which help cancer cells invade surrounding tissues.
d. Enhancing Radiation Sensitivity: Curcumin increases radiation effectiveness by causing DNA damage, stopping cell division (cell cycle arrest), and triggering apoptosis (programmed cell death). Additionally, curcumin halts the cell cycle at the G2/M phase, a critical point where cells prepare to divide, making them more vulnerable to radiation therapy [62,80,81,82,83,84,85,86,87,88,89,90,91,92,93,94,95,96,97,98,99,100,101,102,103,104,105,106,107].

### 3.5. Comprehensive Single-Cell Transcriptomics Analysis of Curcumin-Treated Samples: Insights into T-Cell Subset Dynamics

Understanding how the immune system responds to curcumin at a molecular level can help in developing more effective anti-cancer treatments. Studies using single-cell transcriptomics have identified changes in the activation states of different T-cell subsets, such as CD4+ and CD8+, when exposed to curcumin [108]. Single-cell RNA sequencing (scRNA-seq) has further clarified how curcumin influences immune responses within the tumor environment.

Curcumin enhances T-cell activity, leading to increased activation and proliferation. It also interacts with key immune-related cytokines, including CXCL8 and IL1B, which contribute to immune response regulation. The strong binding of curcumin to these cytokines suggests its potential to boost T-cell-mediated anti-tumor effects [108]. The use of scRNA-seq has advanced our understanding of T-cell diversity and immune responses, highlighting the role of curcumin as an immunomodulator [109]. Additionally, the involvement of the Fli-1 transcription factor in T-cell activation further supports the regulatory role of curcumin [110]. However, effects of curcumin on immune responses may vary depending on the tumor environment and an individual’s immune system. This variability suggests that a personalized treatment approach is necessary to maximize the effectiveness of curcumin as an anti-cancer therapy.

### 3.6. Potential Effects of TTN Mutations on Curcumin Metabolism: Insights into CYP450 Interactions

The TTN gene encodes the titin protein, which is crucial for muscle structure and elasticity. Mutations in TTN are typically linked to heart and muscle disorders. Recent studies suggest that TTN mutations may indirectly affect curcumin metabolism, which is primarily controlled by cytochrome P450 (CYP450) enzymes. During phase I metabolism, curcumin is oxidized by CYP2C9, CYP1A2, and CYP3A4 enzymes. In phase II metabolism, curcumin undergoes conjugation via UDP-glucuronosyltransferases (UGTs) and sulfotransferases (SULTs), resulting in the formation of glucuronides and sulfates.

Systemic changes caused by TTN mutations could alter CYP450 enzyme activity, potentially affecting curcumin metabolism. This may lead to changes in curcumin bioavailability, slower elimination, and fluctuations in its concentration in the blood, which could either weaken its therapeutic effects or increase toxicity risks. While TTN mutations do not directly regulate CYP450 enzymes, their broader systemic effects may indirectly influence curcumin metabolism. More research is needed to understand the precise impact of TTN mutations on CYP450-related curcumin metabolism.

### 3.7. Existing Curcumin Nano-Delivery Systems in RCC

Nano-delivery systems are advanced drug delivery technologies that improve the stability and bioavailability of therapeutic compounds. These systems enable the targeted delivery of curcumin and other bioactive agents while preserving their effectiveness. By enhancing absorption and ensuring controlled release, nano-delivery systems improve treatment outcomes. Currently available curcumin nano-delivery systems for RCC therapy include metal-based nanocarriers (iron oxide, gold, and silver) that improve curcumin delivery into cells, polymeric nano particles such as chitosan-based carriers that allow targeted tumor cell delivery while maintaining controlled curcumin release, lipid-based nanoparticles (solid lipid nanoparticles and liposomes) that enhance curcumin’s stability and bioavailability, nano-gels and micelles that improve curcumin circulation and solubility, maximizing its therapeutic effects.

This study highlights the importance of identifying curcumin-sensitive genes to better predict patient responses to curcumin-based therapies. In contrast, existing curcumin nano-delivery systems focus on improving curcumin’s pharmacokinetics and tumor-targeting capabilities through nanoparticle formulations. These systems have demonstrated both clinical and preclinical effectiveness in slowing RCC progression. Additionally, our research emphasizes the role of apoptosis regulators (DNMT1, BCL2) and tumor suppressors (DNMT3A, PTEN) in curcumin sensitivity. Current nano-delivery systems work by increasing tumor-specific curcumin accumulation, improving cellular uptake via receptor-mediated pathways, and enabling the sustained release of curcumin within tumors. While our study does not focus on curcumin bioavailability, existing nano-delivery approaches address this challenge by enhancing curcumin’s stability, solubility, and targeted delivery. Combining nano-drug delivery systems with knowledge of curcumin-sensitive genes may improve the clinical effectiveness of curcumin, leading to more personalized treatments for kidney cancer (KIRC and KIRP) patients. Future research should integrate biomarker-based therapeutic strategies with curcumin-loaded nano-delivery systems to optimize treatment outcomes for these patients.

### 3.8. Future Directions

The result of the progression-free survival (PFS) analysis (Figure 1C) yielded a near-significant *p*-value (0.0512), suggesting that PTEN and DNMT3A expression variations may influence PFS outcomes. However, as the conventional threshold for statistical significance is *p* ≤ 0.05, our findings do not fully meet this criterion. Further research with larger sample sizes, mechanistic studies, and refined patient stratification is needed to clarify the role of these curcumin-sensitive genes in prognostic outcomes for KIRC and KIRP patients. These studies could also provide insights into their potential impact on curcumin-based therapies. Although the correlation analysis between curcumin-sensitive genes and overall survival (OS), disease-specific survival (DSS), and disease-free survival (DFS) did not yield statistically significant results, these genes may still play a role when considered alongside other molecular factors and patient subgroups. Further research incorporating functional studies, diverse tumor types, molecular subtypes, and treatment regimens could enhance a better understanding of their influence on OS, DSS, DFS, and PFS in KIRC and KIRP patients.

Oncoprint analysis (Figure 1E) indicates that PTEN exhibits the highest genetic alterations (3%), including deep deletions, truncating mutations, splice mutations, and missense mutations, significantly reducing the overall survival of KIRC patients over KIRP patients. These findings suggest that genetic modifications in regulatory genes such as PTEN, DNMT1, BCL2, and MIR-148/148 may influence prognosis and curcumin-related treatment responses. Further studies are necessary to explore the molecular mechanisms behind these differences and their clinical implications.

Despite the promising therapeutic potential of curcumin, its clinical application remains limited due to poor bioavailability. Rapid metabolism and systemic elimination hinder its effectiveness. Strategies to enhance the bioavailability of curcumin include nanoparticle formulations, liposomes, micelles, and solid lipid nanoparticles, which improve stability, solubility, and circulation time. Additionally, adjuvants like piperine (from black pepper) inhibit curcumin metabolism, increasing its bioavailability. Ongoing research also explores combining curcumin with conventional cancer therapies to enhance treatment efficacy, reduce toxicity, and improve patient quality of life.

There is a notable lack of research on the PI3K/AKT signaling pathway in tRCC, a rare kidney cancer subtype. Investigating the presence and function of this pathway in tRCC is essential, alongside experimental studies on the effects of curcumin on this pathway, which could inform novel therapeutic strategies. KIRC, KIRP, and tRCC have distinct molecular and transcriptomic characteristics that influence their oncogenesis and clinical features. KIRC pathogenesis is driven by alterations in the PI3K/AKT/Wnt-β/mTOR pathways, miRNA hypermethylation, and metastasis-related signatures (COL1A2 and POSTN). tRCC is characterized by ceRNA, circRNAs, and lncRNAs, while KIRP’s transcriptomic mechanisms are less understood, necessitating further research.

Chemokines enhance immune responses by attracting immune cells to the tumor microenvironment, particularly in KIRC compared to KIRP and KICH [111,112,113]. The development of chemokine activators may improve immune response and cancer cell destruction in KIRP, ultimately intensifying immune activity in KIRC patients.

The identification of the top 10 most frequently mutated genes in KIRC and KIRP (Figure 6) is a significant finding, with TTN being the most frequently mutated in both tumor types. Investigating the signaling axis of TTN could yield valuable insights for developing an effective dual treatment for KIRC and KIRP. TTN shows similar expression levels in KIRC and KIRP; although slightly higher in KIRC than in KIRP (Figure 6), it is not an absolute diagnostic marker for KIRC. Additional studies using molecular markers such as VHL or PBRM-1 are necessary for diagnostic confirmation. Genes associated with KIRC in the drug target network (e.g., DNAJA2, DNAJB1, HSP90AA1, HSPA1B, HSPA1L, HSPA4, DNAJA1, DNAJA2, DNAJA4, HSPA8, STIP1, and HSPA2) play a role in antigen presentation and tumor suppression. Developing pharmacological agents that activate these genes could revolutionize KIRC treatment.

HIFA/HIFB proteins drive tumor-promoting growth factors such as VEGF, while STAT3 enhances PD-L1 transcription, facilitating immune evasion by targeting immune cells for apoptosis. Developing drugs that block these transcription factors from binding to their protein targets could be a breakthrough in anti-KIRC therapy. In summary, curcumin shows promise as a complementary cancer therapy due to its multifaceted anti-cancer mechanisms and potential to enhance conventional treatments. While bioavailability remains a challenge, innovative drug delivery systems are improving its therapeutic application. Future clinical trials and translational studies will be essential to fully realize the potential of curcumin in renal cancer treatment.

## 4. Materials and Methods

### 4.1. Study Selection

The cBioportal repository of gene expression datasets, a reputable and widely utilized platform, was searched on 12 January 2025, utilizing the keyword “renal”. This search yielded an impressive array of 19 different primary data, encompassing a staggering total of 3795 RCC patient samples. However, in order to ensure the utmost relevance and specificity of the dataset, studies that did not pertain to renal clear cell, translocation, or papillary cell carcinomas were diligently excluded. This rigorous selection process resulted in the identification of 6 studies, comprising a total of 1123 patients’ clinical samples. Further refining the dataset, an additional 2 studies focusing exclusively on renal clear cell carcinoma and papillary cell carcinomas were chosen, ultimately providing a robust dataset consisting of 919 RCC patient samples. Additionally, within this subset, a screening process was conducted to identify samples suitable for somatic mutation analysis, resulting in the inclusion of 797 samples for further investigation.

In order to comprehensively explore the relationship between immunotherapy and radiation response, the previously obtained dataset of 919 samples was subjected to an additional screening process. This screening involved the selection of patients who had received pre/post radiation, pre/post sorafenib, and pre/post bevacizumab treatments. As a result, a highly pertinent and representative subset of 82 public transcriptome data samples of KIRC and KIRP patients was obtained, enabling a more nuanced analysis of the immunotherapy and radiation response studies.

#### Search Strategy

From the utilization of carefully selected keywords, a total of four databases, including Scopus, Pubmed, MEDLINE, CINAHL, Web of Science, and EMBASE, were consulted to yield an impressive number of 4027 studies that proved to be of utmost relevance to our research endeavor. After sifting through these studies, a comprehensive screening process was undertaken, resulting in the identification of 3720 abstracts worthy of further consideration. The elimination of 307 duplicate studies based on their titles ensured a refined pool of materials for further examination. Subsequently, 3681 papers were excluded following a thorough evaluation of their abstracts. Nonetheless, the remaining 24 papers were not discarded hastily but were instead subjected to a review. Out of this discerning analysis, it became apparent that 18 articles failed to meet the necessary exclusion and inclusion criteria, prompting their removal from our investigation. This discerning approach left us with a final selection of just six studies, which we have chosen to include in our work.

To augment the comprehensiveness and robustness of the study, additional references were diligently identified from the bibliographies of the retrieved studies. This process ensured that no pertinent studies or sources were overlooked, thereby enhancing the completeness and reliability of the findings. To ensure the validity of the findings, an extensive and thorough literature search was executed, encompassing a wide range of reputable databases and sources such as Scopus, Pubmed, MEDLINE, relevant systematic reviews, CINAHL, Web of Science, and EMBASE. Additionally, in order to obtain a comprehensive and robust dataset, public genomic libraries and databases, including cBioportal, were extensively utilized to source patients’ genomic primary clinical data, with a specific focus on renal cancer.

### 4.2. Inclusion Criteria

To ensure the validity and reliability of the findings, strict inclusion criteria were established. The study population specifically comprised adults aged 18 years and above who were diagnosed with renal cancer at various metastatic stages. To maintain the highest level of precision and relevance, only patients diagnosed with kidney renal clear cell carcinoma (KIRC) and kidney renal papillary cell carcinoma (KIRP) were considered for the cross-sectional studies. Moreover, the inclusion criteria encompassed prospective observational studies, case–control studies, and cross-sectional studies that focused on studying the differentially expressed genes (DEGs) in the renal tissue of renal cell carcinoma patients. To ensure data homogeneity and comparability, only studies that utilized next-generation sequencing methods for the identification of DEGs were deemed suitable for inclusion. As a result, a selection process was conducted, resulting in the inclusion of prospective observational studies, case–control studies, and cross-sectional studies that investigated DEGs in the kidney renal clear cell and papillary carcinoma patients’ tissues, further bolstering the robustness of the study.

### 4.3. Exclusion Criteria

Specific criteria were applied for including and excluding patient samples to ensure accurate result interpretation. Samples with transcript per million (TPM) read counts below 1 in more than 50% of genes were excluded, as TPM values under 1 indicate poor sequencing quality, uninformative data, and low RNA expression. Samples with somatic mutation sequencing depths below 20× or allele mutation frequencies under 5% were also excluded. Patients with a follow-up period of less than 30 days were excluded to avoid bias from early patient death. Samples with excessive missing data or duplicates were excluded. Copy number alterations (CNAs) were assessed using the GISTIC 2.0 tool, and only significant CNAs (Log2 fold change > ±0.2) were included, while those with lower amplification values (Log2 fold change < ±0.2) were excluded. Intervention studies focusing on curcumin and renal cell carcinoma (RCC) utilizing blood and non-renal papillary and clear cell carcinoma tissues have been deliberately excluded. Similarly, studies lacking primary data, such as conference proceedings, case reports, narrative review articles, and editorials, were omitted. 

### 4.4. Screening of Articles for Eligibility

In the initial phase, an exhaustive screening of articles from various sources was conducted, carefully eliminating articles with irrelevant titles and duplicates to ensure the utmost accuracy and relevance of the study. Subsequently, during the second phase, a thorough examination of abstracts from the screened articles was conducted, eliminating those that did not align with the predefined inclusion criteria. Finally, in the third phase, a comprehensive screening of the articles from the second phase was executed to exclude in vitro and in vivo studies, systematic reviews, meta-analyses, and any article that deviated from the established inclusion criteria. It is imperative to note that all authors actively participated in both the screening and data extraction phases, further ensuring the meticulousness and integrity of the research.

### 4.5. Study Quality

To ensure the highest level of scientific rigor and credibility, the renowned Joanna Brigs Institute critical appraisal tools were employed to independently examine the study quality. MHE and NAH have meticulously assessed the obtained results, and these results have been further validated by the same MHE and NAH. A meticulous scoring system was implemented, whereby an overall score above 69% was deemed a high-quality paper with a low risk of bias. Scores ranging between 50% and 69% were categorized as moderate risk of bias, while scores below 50% were classified as high risk of bias and low-quality papers.

### 4.6. Data Extraction, Processing and Analysis

To maintain the utmost rigor and precision in our study, only data that strictly adhere to the inclusion criteria were extracted by the four diligent review authors (F.E.E., Y.S.N., I.K.U, and M.O.A) who actively participated in the data extraction process. The data collection and extraction were independently standardized using a comprehensively designed data collection form. This form was instrumental in gathering essential information on study results, such as *p*-values, overall survival, genomic alteration fraction, and mutation count. Additionally, crucial study characteristics, including the number of patients, study design, names of authors, and year of publication, were recorded. Furthermore, patients’ demographics, including age, ethnicity, gender, and cancer stage, were carefully incorporated into the standardized data extraction form.

### 4.7. KIRC and KIRP Survival Analysis

Survival analysis was performed using clinical patient data from the TCGA database via cBioPortal. The Kaplan–Meier method was employed to generate survival curves, while the log-rank test was used to evaluate statistical significance (*p*-value) in relation to curcumin-sensitive genes. Additionally, OncoPrint analysis was conducted to examine the correlation between overall survival (OS) outcomes in KIRC and KIRP patients and alterations in curcumin-sensitive genes.

### 4.8. KIRP and KIRC Differential Expression Analysis

A comprehensive differential expression analysis was conducted on tissue samples from KIRC and KIRP patients who had undergone sorafenib, bevacizumab, and radiation therapies. This analysis utilized primary clinical data from the TCGA database and was performed using Python 3.12.5 and R. 4.4.2 By examining tissue samples collected both before and after treatment, the study provided detailed insights into the impact of these therapies on gene expression.

### 4.9. Construction and Analysis of PPI Network

To unravel the intricate interplay between proteins, String bioinformatics software (https://string-db.org accessed on 28 May 2025) was judiciously employed for protein–protein interaction (PPI) network analysis. With a stringent threshold of 9.0, this analysis facilitated the identification of crucial nodal connectivity. Only nodes with a connectivity above 50 were deemed significant for further analyses, as they serve as pivotal drug-targeted genes that are intricately associated with both KIRC and KIRP.

#### 4.9.1. Drug-Targeted Gene Analysis

The STITCH Bioinformatics database was utilized in order to conduct a thorough screening for potential drug candidates. This screening process involved the utilization of both KIRC and KIRP hub genes, as well as drug-targeted genes that were obtained from the protein–protein interaction (PPI) network analysis. By employing this approach, we were able to identify potential drugs that could be targeted toward the treatment of KIRP and KIRC.

#### 4.9.2. Gene Ontology

In order to gain a deeper understanding of the role that candidate genes play in the associated pathways of KIRP and KIRC, gene ontology (GO) and Kyoto Encyclopedia of Genes and Genomes (KEGG) pathway analyses were conducted on the top 100 altered VHL and TTN neighboring genes. This analysis was performed using the cBioportal for cancer genomics on primary data of patients. Additionally, the cBioportal and R package were also employed to carry out differential expression and functional analysis with VHL and TTN. These methods allowed for a comprehensive examination of the differential expression patterns and functional characteristics of these genes.

#### 4.9.3. KIRC and KICH Mutation Study

The analysis of frequently mutated gene expression in primary patient clinical samples was conducted using the TCGA database.

## 5. Conclusions

This study investigates the association between curcumin-sensitive genes and patients’ survival outcomes in the various subtypes of renal cell carcinoma (RCC), as well as how these genes respond to different standard treatment options. The results showed that these genes had no significant effect on patients’ overall survival (OS), disease-free survival (DFS), or disease-specific survival (DSS). However, there was a slight trend suggesting they may help slow disease progression. Additionally, oncoprint analysis indicated that KIRP patients with mutations in MIR-148/148, DNMT1, and PTEN had better overall survival than KIRC patients with the same mutations. The combination of curcumin and sunitinib showed encouraging potential in overcoming drug resistance in clear cell renal cell carcinoma (ccRCC). mRNA expression analysis revealed a lack of significant response of curcumin-sensitive genes to the effects of standard treatments like bevacizumab, radiation, sorafenib, and sunitinib. However, when curcumin was combined with sunitinib, these genes showed a significant response, suggesting that curcumin may enhance the effectiveness of sunitinib and other conventional therapies.

## Data Availability

The data presented in this study are openly available in the cBioportal curated from the TCGA database at cBioPortal for Cancer Genomics.

## Abbreviations

DEGs	Differentially Expressed Genes
KIRC	Kidney Renal Clear Cell Carcinoma
KIRP	Kidney Renal Papillary Cell Carcinoma
KICH	Chromophobe Renal Cell Carcinoma
RCC	Renal Cell Carcinoma
TCGA	The Cancer Genome Atlas
GO	Gene Ontology
ssGSEA	Single Sample Gene Set Enrichment Analysis
PPAR	Peroxisome Proliferator Activated Receptor
JAK-STAT	Janus Kinase/Signal Transducers and Activators of Transcription
Rap-1	Ras-proximate-1 or Ras-related protein 1
cGMP-PKG	Cyclic Guanosine Monophosphate dependent Protein Kinase
DEGs	Differentially Expressed Genes
PTEN	Phosphatase and Tensin homolog
DNMT3A	DNA (cytosine-5)-Methyltransferase 3A
NCOA4	Nuclear receptor Coactivator 4
FTH1	Ferritin Heavy chain
BCL2	B Cell Lymphoma 2
MIR-148/148	Micro RNA 148
CYP450	Cytochrome P450
P13K/AKT	Phosphatidylinositol 3-kinase
